# Chemical Composition and Antimicrobial Effectiveness of *Ocimum gratissimum* L. Essential Oil Against Multidrug-Resistant Isolates of *Staphylococcus aureus* and *Escherichia coli*

**DOI:** 10.3390/molecules24213864

**Published:** 2019-10-26

**Authors:** Ramaiana Soares Melo, Águida Maria Albuquerque Azevedo, Antônio Mateus Gomes Pereira, Renan Rhonalty Rocha, Rafaela Mesquita Bastos Cavalcante, Maria Nágila Carneiro Matos, Pedro Henrique Ribeiro Lopes, Geovany Amorim Gomes, Tigressa Helena Soares Rodrigues, Hélcio Silva dos Santos, Izabelly Linhares Ponte, Renata Albuquerque Costa, Gabriel Sousa Brito, Francisco Eduardo Aragão Catunda Júnior, Victor Alves Carneiro

**Affiliations:** 1 Center of Bioprospection and Experimentation Molecular Applied (NUBEM), University Center INTA–UNINTA, Sobral, Ceará 62050-100, Brazil; 2 Laboratory of Biofilms and Antimicrobial Agents, Federal University of Ceará, Sobral, Ceará 62042-280, Brazil; 3 Center of Exact Sciences and Technology, State University Vale do Acaraú, Sobral, Ceará 62040-370, Brazil; 4 Pharmaceutical Coordinator of the Laboratory of Clinical Analysis of Santa Casa de Misericórdia de Sobral, Sobral, Ceará 62010-550, Brazil; 5 Center for Exact, Natural and Technological Sciences, State University of the Tocantina Region of Maranhão, Imperatriz, Maranhão 65901-480, Brazil

**Keywords:** antimicrobial activity, multiresistant microorganisms, ciprofloxacin, oxacillin, MDR bacteria, antibiofilm activity

## Abstract

The study investigated the antimicrobial activity of the essential oil extract of *Ocimum gratissimum* L. (EOOG) against multiresistant microorganisms in planktonic and biofilm form. Hydrodistillation was used to obtain the EOOG, and the analysis of chemical composition was done by gas chromatography coupled with mass spectrometry (GC/MS) and flame ionization detection (GC/FID). EOOG biological activity was verified against isolates of *Staphylococcus aureus* and *Escherichia coli*, using four strains for each species. The antibacterial action of EOOG was determined by disk diffusion, microdilution (MIC/MBC), growth curve under sub-MIC exposure, and the combinatorial activity with ciprofloxacin (CIP) and oxacillin (OXA) were determined by checkerboard assay. The EOOG antibiofilm action was performed against the established biofilm and analyzed by crystal violet, colony-forming unit count, and SEM analyses. EOOG yielded 1.66% *w*/*w*, with eugenol as the major component (74.83%). The MIC was 1000 µg/mL for the most tested strains. The growth curve showed a lag phase delay for both species, mainly *S. aureus*, and reduced the growth level of *E. coli* by half. The combination of EOOG with OXA and CIP led to an additive action for *S. aureus*. A significant reduction in biofilm biomass and cell viability was verified for *S. aureus* and *E. coli*. In conclusion, EOOG has relevant potential as a natural alternative to treat infections caused by multiresistant strains.

## 1. Introduction

The rise of infections caused by multidrug-resistant (MDR) bacteria has become a worrying public health problem [1]. The occurrence of these strains is frequently related to strong selective pressure caused by indiscriminate and inadequate use of antibiotics in hospitals [2]. However, this is not a problem restricted to a nosocomial environment, as it can also be related to infections caused by foodborne pathogens [3]. In addition, another important issue is the ability of several bacteria to form a complex multicellular structure, called biofilms [4]. These tridimensional communities are built mainly by bacterial cells embedded into extracellular polysaccharides secreted by themselves [5], and this arrangement reduces the effectiveness of antimicrobial agents [6,7]. Thus, antibiotic resistance reduces the number of therapeutic options and causes an increase in hospitalization costs/time of patients, thus increasing the morbimortality rates [8].

As an alternative therapeutic source, essential oils (EOs) extracted from plants are qualified as an important biotechnological product to be explored for pharmaceuticals and food companies [9,10,11,12]. EOs are constituted by volatile organic products of various classes, such as aldehydes, terpenes, and phenolic compounds, synthesized by a secondary metabolism from different parts of plants [13]. Many studies have documented EOs to be effective antimicrobial agents against several pathogens [14,15,16]. Usually, this EO effect is closely related to the capability of permeabilizing and/or destroying membrane integrity, leading to the leakage of intracellular substances [17,18,19]. Moreover, since the last decade, the antibiofilm activity of EOs has been widely explored and reported in the literature [20,21,22,23].

*O. gratissimum L.* (EOOG), or clove basil, belongs to the Lamiaceae family, and it is commonly found in Africa, Asia, and South America [24]. This species is included among the 71 plants described in the National List of Medicinal Plants of Interest to the Unified Health System (RENISUS), created in 2009 in Brazil [25]. The use of leaf infusion has already been reported in folk medicine to treat fever, flu, and kidney problems [26]. Other reports have shown that the chemical composition of EOOG depends on the climate zone and soil type wherein the species is cultivated [27], and this feature can influence biological activity, since the major constituents can be altered [28]. Among the biological applications of EOOG, the antimicrobial properties have been studied separately or associated with conventional antibiotics, reestablishing the sensitivity of MDR bacteria [29,30,31]. Therefore, the aim of this study was to characterize the chemical constitution of EOOG extracted from dried leaves and explore in vitro the antibacterial and antibiofilm activity against *S. aureus* from clinical samples and *E. coli* isolates from fresh tilapia fillets commercialized in the retail trade of Sobral, Brazil, both of which having multiresistance phenotypes.

## 2. Results

### 2.1. Extraction and Chemical Composition of the EOOG

The dried leaves of *O. gratissimum* L. submitted to hydrodistillation in a Clevenger apparatus showed an EO yield of 1.66% (*w*/*w*). The chemical composition of the EOOG and their respective Kovats indices (KIs) are listed in Table 1.

**Figure 1:**
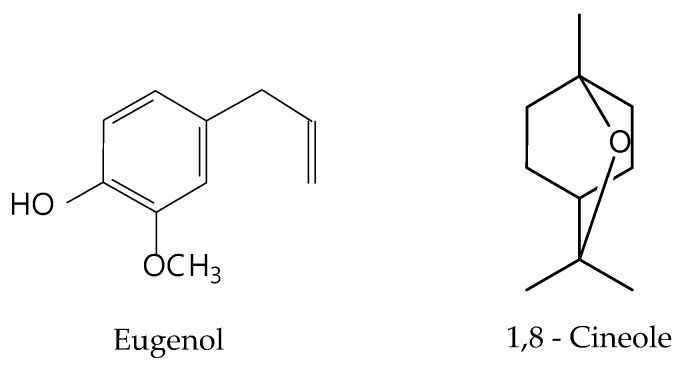
Chemical structures of the major constituents from the EO of *Ocimum gratissimum* L. (EOOG) dry leaves.

Analysis by gas chromatography coupled with mass spectrometry (GC/MS) and flame ionization detection (GC/FID) resulted in the identification of 19 compounds in the sample, representing 99.42% of the constituents. The most abundant volatile component was eugenol (74.83%), followed by 1,8-cineole (15.16%) (Figure 1). However, several classes of chemical compounds were found, mainly phenylpropanoid (74.83%), oxygenated monoterpenes (16.09%), hydrocarbon sesquiterpenes (6.96%), and minor amounts of hydrocarbon monoterpenes (0.92%) and oxygenated sesquiterpenes (0.62%).

### 2.2. Antibacterial Activity

The results demonstrated that EOOG has significant antimicrobial activity against all tested strains. The EO had a large inhibition zone diameter (IZD) against *S. aureus* cells, among which, 5B and 2B had higher (20 mm) and lower (14 mm) IZD, respectively. However, this promising effect decreased slightly against *E. coli* cells, and the IZD values were kept around 12–13 mm. According to the microdilution method, the minimum inhibitory concentration (MIC) of EOOG was observed at 1000 µg/mL indistinctly for *S. aureus* and *E. coli* cells, with the exception of 5B, which had twice the amount, 2000 µg/mL. It is important that salient *E. coli* strains had the same bacteriostatic and bactericidal concentration levels (MIC and MBC), at 1000 µg/mL. Therefore, *S. aureus* strains presented double the quantity (2000 µg/mL) necessary to eliminate the cells (2B, 5B, and 7B). All IZDs, as well as MIC and MBC values of EOOG, are shown in Table 2.

### 2.3. Bacterial Growth Curve

**Figure 2:**
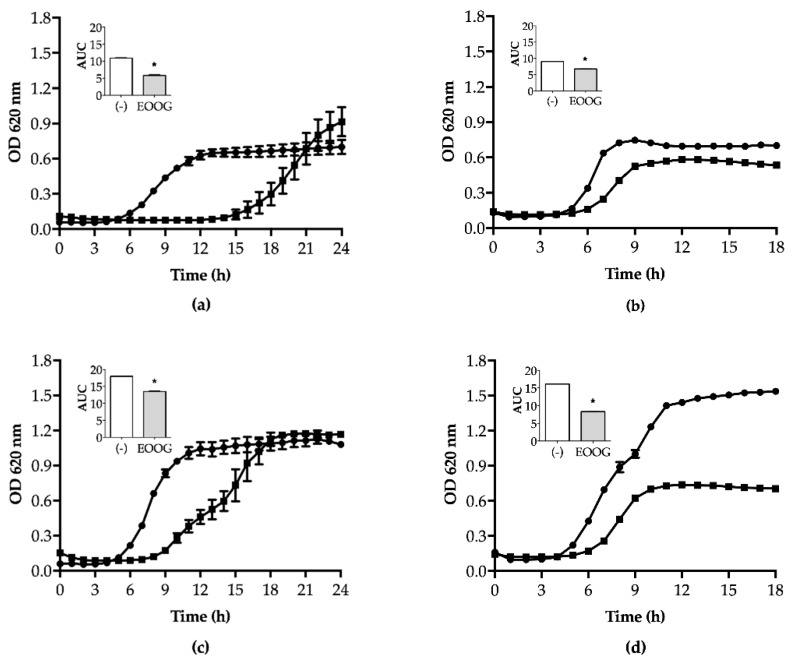

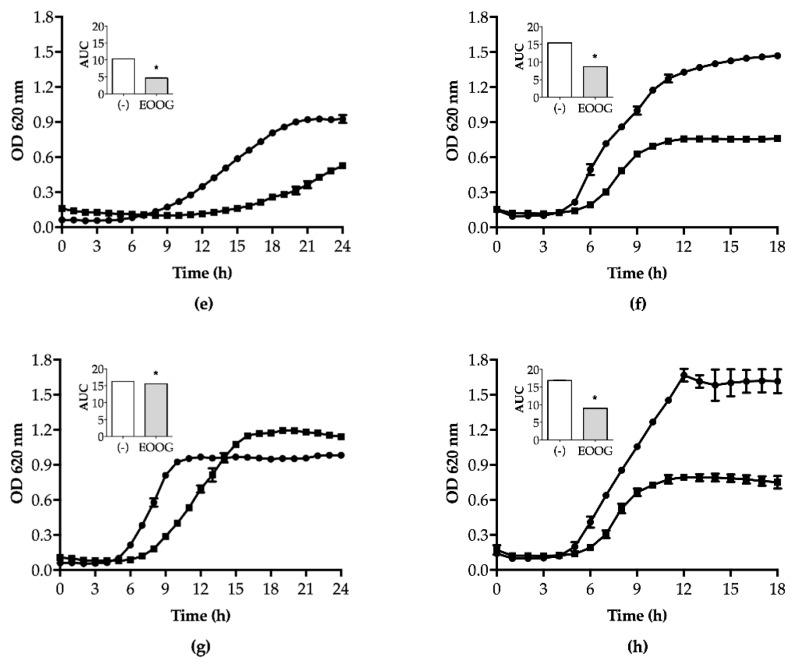
Effect of EOOG (1/2 MIC) on the bacterial growth curve (line graph) of *S. aureus* (**a**) ATCC 6538, (**c**) 2B, (**e**) 5B, and (**g**) 7B and of *E. coli*, (**b**) ATCC 11303, (**d**) P12, (**f**) P25, and (**h**) P36. The growth level of the EOOG-treated (■/grey bar) and -untreated (●/white bar) cells was quantified by the area under the curve (AUC). * Statistically different by ANOVA (*p* < 0.01) compared to the control group.

The majority of strains in the control group, media broth only, clearly presented different growth, adaptation (lag), exponential (log), and stationary phases, as expected. However, the presence of EOOG (1/2 MIC) interfered with the performance of these microorganisms during curve development, as shown in Figure 2.

*S. aureus* cells left the lag phase after 5 h of incubation, which was followed by a 5 h exponential phase before reaching the stationary stage, which consisted of 12 h of development. The presence of EOOG delayed the beginning of the multiplication step, prolonging the lag phase. However, at the end of growth (24 h), the optical density (OD) was similar between the control and the EOOG group. However, *S. aureus* 5B reached the stationary phase only after 21 h of incubation. The EOOG reduced the bacterial growth to 50% of the control OD at the end of the curve (Figure 2).

All *E. coli* displayed three growth phases after 18 h. Except for the ATCC strain, they presented a short lag and an expressive log growth, reaching a high level (>1,500 UA) of absorbance at the end of the curve. The influence of EOOG on *E. coli* strains caused a reduction in cell density, which was determined by OD values. The establishment of the stationary phase was at least 50% below the control curve, except for the ATCC strain, which had a 20% reduction only. The growth level was determined by the area under the curve (AUC), and the treated groups showed a significant reduction (*p* < 0.01) for all tested strains, as shown by bar graphs in Figure 2.

### 2.4. Combination between EOOG and Antibiotics

The 5B and P12 strains, which presented resistance profiles by the VITEK^®^2 system (BioMérieux, Marcy-l’Étoile, France), were used to evaluate the EOOG and antibiotics interaction performed by checkerboard assay. *S. aureus* (5B) and *E. coli* (P12) obtained the same MIC for ciprofloxacin (CIP), 62.50 µg/mL. The OXA bacteriostatic concentration was determined only against 5B, which was 2000 µg/mL, since this kind of antibiotic is not clinically recommended to treat *E. coli*-related illnesses.

The combination of the EOOG with antibiotics, CIP and OXA had fractional inhibitory concentration index (FIC*i*) of 0.516 and 0.562, respectively, showing additive effect interaction between them (Table 3). In both cases the combination with antibiotics allowed to decrease by half the EO concentration, only. Furthermore, the CIP and OXA doses were 16- and 64-fold less, respectively, than individual use. In relation to the P12 strain, the effect of the EOOG/CIP combination was antagonistic (FIC*i* = 2). The individual and combined MIC of the EO and antibiotic, as well as the FIC*i* interpretation and reduction in drug concentration, are shown in Table 3.

### 2.5. Activity on Preformed Biofilm

**Figure 3:**
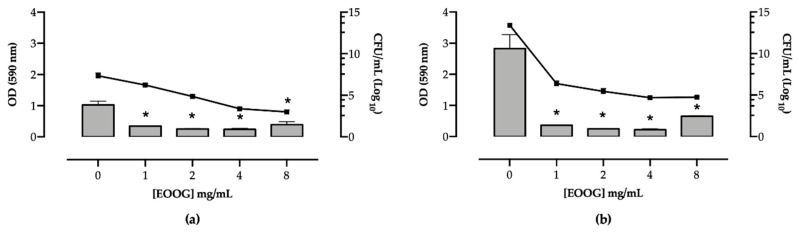
Antibiofilm activity of different concentrations of EO of *Ocimum gratissimum* L. against preformed biofilms of *S. aureus* 5B (**a**) and *E. coli* P12 (**b**), respectively. Biomass quantification by crystal violet staining (OD 590 nm, bars) and cell enumeration by colony count (log_10_ CFU/mL, lines). * Statistically different by ANOVA (*p* < 0.01) compared to untreated cells.

The EOOG activity against the biofilm of *S. aureus* (5B) and *E. coli* (P12), pre-established for 24 and 12 h, respectively, was evaluated according to biomass by the crystal violet method and cell enumeration by CFU count (Figure 3).

The strains tested have an equally high capacity for biofilm formation. However, *E. coli* produced 2.75-fold more biomass and an additional six log units of viable cells into the biofilm than *S. aureus*, even when less time (12 h) is given for biofilm development. After EOOG treatment (4 h), these features decreased significantly for both 5B and P12 strains. At a lower concentration, 1 mg/mL, the EOOG-treated biofilm presented a great reduction, mainly for the *E. coli* strain, which decreased the biomass by 12-fold, and declined in the number of viable cells in the biofilm by eight log_10_ units. According to *S. aureus*, these values were discretely lower, the biomass reduced 4-fold, and the CFU count decreased by four log_10_ units. In both strains, the EOOG was unable to eradicate the biofilm, even at a higher concentration, where some remaining viable cells were detectable.

### 2.6. Scanning Electron Microscopy

**Figure 4:**
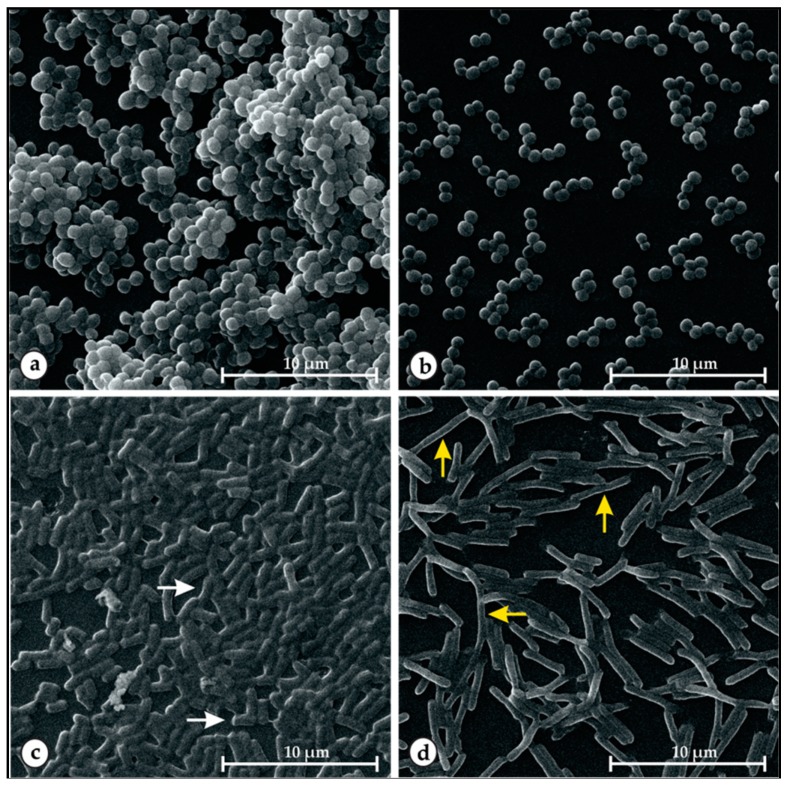
Scanning electron microscopy images of preformed biofilms of *S. aureus* (5B) and *E. coli* (P12) treated with the EO of *Ocimum gratissimum* L. (EOOG) at MIC doses. Untreated 5B (**a**) and P12, (**c**) and EOOG-treated 5B, (**b**) and P12 (**d**) cells. In the control condition, *E. coli* presented rod-shaped (white arrow) cells, and after EOOG treatment, the P12 cells displayed an elongated (yellow arrow) morphology status.

The preformed biofilm structure of *S. aureus* 5B (24 h) and *E. coli* P12 (12 h), treated and untreated with EOOG, were evaluated through scanning electron microscopy (SEM). Comparative analysis of SEM showed a significant reduction in cell density in the biofilm after 4 h of EOOG treatment when compared to control conditions in both strains (Figure 4).

In control groups, it is possible to verify cell clusters forming a well-defined three-dimensional structure, mainly for 5B. EOOG treatment at MIC doses was able to disrupt the bacterial community of both strains, reducing the number of adsorbed cells in the biofilm. EOOG-treated P12 cells were clearly found elongated compared to the cells in the control condition, as shown in Figure 4c,d, respectively.

## 3. Discussion

In the present study, it was found that dried leaves of *O. gratissimum* L. collected from Alto da Pipira, located in Maranhão State, Brazil, obtained yield of 1.66% *w*/*w*. Similar amount (1.12% *w*/*w*) was extracted from dry aerial parts of *O. gratissimum* cultivated in Londrina, State of Paraná, Brazil [33]. Yield of 0.21% *w*/*w* was also found using fresh leaves and branches obtained from Uganda, East African country [34]. Thus, the type of raw material used for extraction process, dry or fresh samples, may have impact on the essential oil yield. It was possible to identify 99.42% of the constituents, corresponding to 19 compounds, among which, eugenol (74.83%) and 1,8-cineole (15.16%) were the most abundant in the EOOG composition (Table 1). Other studies have also reported eugenol as the main constituent of this oil; however, the second most present component has been different, such as methyl eugenol, germacrene-D [35], terpinolene [36], citronellal [37], and *α*-ocimene [38].

It has been proven that vegetative stages can influence the constitution of EOs [39]. EOOG obtained in the Republic of Benin, West Africa, showed a difference in the quantification of prevalent constituents in relation to vegetative, pre-flowering, and flowering stages. In addition, diverging from the findings in the present research, other major constituents, *p*-cymene and thymol, were detected [40]. However, as seen above, a range of variables, such as seasonality, circadian rhythm, temperature, water availability, incidence of ultraviolet radiation, nutrient availability, altitude, and pathogen attack to plants, can interfere with a plant’s secondary metabolism and can considerably modify the OE composition [41].

Currently, due to the emergence and spread of MDR bacteria, it is necessary to search for new alternatives to combat this kind of pathogen, and to reverse the deficiency of antibiotic options against these infections [42]. The present investigation corroborated the promising applications of EOs as an alternative therapy against bacterial isolates, such as *S. aureus* and *E. coli*. Initially, the EOOG antimicrobial activity was accessed by disk diffusion and microdilution techniques to verify the sensibility profile of the tested strains. Thus, EOOG was effective mainly at concentrations between 1000 and 2000 µg/mL. However, different EOs or chemotypes from the same EO may have a wide MIC variety (from 50 to 6400 μg/mL) against foodborne pathogens [43].

Although the literature has shown Gram-negative bacteria to be more resistant to EOs compared to Gram-positive [44,45], it was possible to verify that EOOG had a greater effect against *E. coli* than *S. aureus* strains, as also demonstrated by the SEM images (Figure 4). This differential EO action on microbial species was also observed in the bacterial growth curve under sub-MIC EOOG exposure. The treated cells clearly had a lag phase extended compared to the control. However, the exponential growth was recovered, and *S. aureus* easily reached optical density measurements similar to the control, while *E. coli* had its growth level reduced by half, as shown by the AUC graphs in Figure 2.

The microbial behavior in response to EO exposure could be due to several reasons, one of which is the ability of small molecules, such as eugenol and 1,8-cineole (Figure 1), major compounds of EOOG, to interact with the outer surface of the cells [44,46,47]. In addition, some bacteria have an adaptive capacity to protect itself against the stress caused by OE attacks that alter the fatty acid profile of the cytoplasmic membrane [48,49]. Therefore, the main bactericidal action of the EO is attributed to the ability to interact with the cell membrane, altering its permeability, causing extravasation of intracellular constituents, and resulting in bacterial death [50]. Furthermore, EOs are made up of various compounds, and it is still believed that this makes it difficult to develop bacterial resistance when compared to antibiotics that have only one cell target, suggesting that EOs can be used to fight MDR bacteria [51].

Natural products also fit as components of interest for combinatorial therapies against MDR bacterial infections [52,53]. Previous studies have reported interactions between EO constituents and conventional antibiotics against *E. coli* MDR [54,55,56]. Oliva et al. (2018), using terpinen-4-ol-rich EO of *Melaleuca alternifolia*, found synergistic activity with OXA against methicillin-resistant *S. aureus* (MRSA) [57]. This effect might be attributed to the ability of the EO compound, such as eugenol/thymol, to increase the antibiotic permeation into the bacterial cell [55,58]. Although many researchers seek to demonstrate a synergism between EO and antibiotics [59,60,61,62], it should be considered that combinations that result in additive effects may be as effective as synergistic effects because, with minor amounts of antibiotics, they provide satisfactory effects [63], as shown by the 16- and 64-fold decreases in CIP and OXA concentrations, respectively, against the 5B strain. Concerning the P12 strain, EOOG combined with CIP was unable to alter the strain resistance, showing an antagonistic effect. However, EOOG in combination with other antibiotics should not be ruled out, since the majority EOOG compound (eugenol) has been explored in previous studies and has demonstrated a synergistic effect using penicillin and colistin [49,54].

Another applicability of EOs is related to biofilm disruption [64]. Since biofilm formation is a virulence factor for many microorganisms, such as *S. aureus* and *E. coli*, and increases tolerance to antimicrobial drugs, new strategies to combat them are sought [65,66]. Budzyńska et al. (2017) described the antibiofilm activity of EOs from cloves, which have a high eugenol percentage (86.2%), and were able to reduce *S. aureus* mono- and dual-species with a *Candida albicans* biofilm [67]. In other case, the EO from bay leaves, cloves, and berry peppers with more than 60% eugenol in their constitution show important activities against antibiotic-resistant biofilms of *E. coli* O157:H7 [68]. Combined with these results, this study shows that eugenol-rich EOOG is able to reduce biomass and the number of viable cells in biofilms from *S. aureus* and *E. coli* MDR.

The mechanism of action against these established cell clusters may be through the disruption of 3D structures and/or the direct killing of the bacteria in the biofilm [69,70,71]. Through SEM images (Figure 4), it can be noted that the cellular damage to *S. aureus* was less significant than that to *E. coli*, since the Gram-negative cell showed a considerable difference in cellular dimensions between EOOG-treated and -untreated bacteria. As shown before, this morphological status could be related to interference in the cell division process due to stress responses, including DNA damage and the inhibition of replication [72,73]. Previous research has also associated antibiofilm activity with the ability of these natural compounds to disturb the bacterial communication mechanism, quorum sensing, and the expression of virulence factors that are responsible for the survival characteristics of the biofilm [74,75,76].

## 4. Materials and Methods

### 4.1. Plant Material

Fresh leaves of *O. gratissimum* L. were collected in the morning at Alto da Pipira (5°26′4.07″ S, 47°17′45.83″ W), in the State of Maranhão, in the northeast region of Brazil. The botanical identification was performed by Prof. Dr. Eduardo Bezerra de Almeida Júnior, and the voucher specimen (No. 11175) was deposited and cataloged in the collection of the Maranhão Herbarium (MAR) of the Department of Biology (CCBS) of the Federal University of Maranhão (UFMA), Maranhão, Brazil.

### 4.2. Essential Oil Extraction

The fresh leaves of *O. gratissimum* were dried in room temperature for four days. Then, the dried material (76.84 g) was mixed with 2.5 L of distilled water and subjected to hydrodistillation in a Clevenger-type apparatus for 3 h to afford a pale yellow oil. The isolated oil, after drying over anhydrous sodium sulfate (Na_2_SO_4_) and filtration, was stored in sealed glass vials and maintained under refrigeration until further analysis. Total oil yield was expressed as a percentage (g per 100 g of dried leaves).

### 4.3. Chemical Composition of EOOG

Qualitative analysis of the chemical composition of the essential oil was performed with a gas chromatograph (GC) coupled with a mass spectrometer (MS), Agilent Model GC-7890B/MSD-5977A (quadrupole), with an electron impact at 70 eV, an HP-5MS methylpolysiloxane column (30 m × 0.25 mm × 0.25 μm, Agilent), a 1 mL/min flowing helium carrier gas, an injector temperature of 250 °C, a detector temperature of 150 °C, and a transfer line at 280 °C. The chromatographic oven was programmed as follows: an initial temperature of 70 °C, with a heating ramp of 4 °C/min to 180 °C, and an increase of 10 °C/min to 250 °C at the end of the run (34.5 min).

Quantitative analysis of the chemical composition of the oil was carried out by GC coupled to a flame ionization detector (FID), Shimadzu Model CG-2010 Plus instrument, with an RTX-5 methylpolysiloxane column (30 m × 0.25 mm × 0.25 μm), a 1:30 flow split injection mode, a 1.00 mL/min flow nitrogen carrier gas, an injector temperature of 250 °C, and a detector temperature of 280 °C. The programming of the chromatographic oven was similar to that used in the GC/MS analysis.

The constituents’ percentages were calculated by the integral area of their respective peaks, related to the total area of all the constituents of the sample. The various constituents of the EO were identified by visually comparing their mass spectra with those in the literature [32] and the spectra provided by the equipment database (NIST11), and by comparing retention rates with those in the literature [32]. A standard solution of n-alkanes (C_9_–C_30_) was injected using the same chromatographic conditions of the sample to calculate the retention index for each peak, as described by Dool and Kratz (1963) [77].

### 4.4. Preparation of Antimicrobial Solutions

EOOG stock solution was prepared in Brain Heart Infusion broth (BHI, Acumedia^®^, Michigan, USA) with 1% tween 80 sterile (VETEC Fine Chemistry LTDA, Rio de Janeiro, Brazil) at 16 mg/mL. From the oil density (1.0074 g/mL), it was firstly mixed EOOG (16 μL) with tween 80 (10 μL) and bring volume to 1 mL with fresh BHI (974 μL). It was considered as control group (untreated cells) in the experimental assays, only broth media plus tween 80 in an equivalent amount for each EOOG concentration tested. Oxacillin sodium (OXA, Blau Pharmaceutical SA, Sao Paulo, Brazil) and ciprofloxacin (CIP, Fresenius Laboratory Kabi, Sao Paulo, Brazil) antibiotics, obtained commercially, were prepared according to manufacture note, and the concentration was adjusted to 32 mg/mL.

### 4.5. Bacterial Strains and Culture Conditions

EOOG activity was evaluated against two representatives, Gram-negative and -positive bacterial cells, four strains of *S. aureus* and *E. coli* species. The standard strains from the American Type Culture Collection (ATCC, USA) were used as sensitive antibiotic strains. The clinical *S. aureus* were isolated and identified in the Laboratory of Microbiology of the Santa Casa de Misericórdia de Sobral, CE, Brazil, as part of the hospital routine. *E. coli* strains were isolated from fresh tilapia fillets (*Oreochromis niloticus*) obtained from the retail trade of Sobral, CE, Brazil [78]. The confirmatory identification and resistance profile of bacterial strains were determined by the VITEK^®^2 system (BioMérieux, Marcy- L’Etoile, France). The source and resistance profile of the microorganisms selected are described below (Table 4).

Separately, cells were cultured from stock culture and maintained at −80 °C in 5 mL of BHI broth at 37 °C overnight before each experimental procedure. Afterward, 50 µL were inoculated into the same culture media (1:10) and grown at 37 °C until late in the exponential phase. Prior to biological assays, the turbidity of bacterial suspension was adjusted against the standard 0.5 McFarland (10^8^ CFU/mL) and diluted with fresh BHI to reach an appropriate cell concentration for each method.

### 4.6. Antimicrobial Activity of EOOG

The antibacterial activity of the EO was firstly determined by the paper disk diffusion method. Briefly, a sterile swab was dipped into each tested bacterial suspension adjusted to 10^8^ CFU/mL in saline, rotated against the tube wall, and rubbed in several directions on the whole surface of Mueller–Hinton agar (MHA) plates (Acumedia^®^, Michigan, USA). Afterward, three disks 6 mm in diameter (Biomérieux, Marcy-l’Etoile, France) and impregnated with 5 µL of pure EOOG were placed equidistantly on the MHA surface. After 24 h of incubation at 37 °C, the antibacterial effect was evaluated by measuring the diameter of inhibitory zones (DIZ) in millimeters, and the results were expressed as means from three determinations.

The minimum inhibitory concentrations (MICs) were determined using a microdilution method following the standard CLSI (Clinical and Laboratory Standards Institute, M07-A10) protocol [79]. Serial two-fold dilutions of 100 µL were prepared with BHI broth in 96-well microtiter plates (KASVI, Paraná, Brazil), obtaining EOOG concentrations from 250 to 8000 μg/mL. For antibiotic CIP and OXA, concentrations ranging from 0.25 to 8000 µg/mL and from 0.25 to 256 µg/mL, respectively, were used. Afterward, 100 µL of the bacterial suspension (10^6^ CFU/mL) were added to each well, yielding a final cell concentration of 5 × 10^5^, and plates were incubated for 24 h at 37 °C. The lowest concentration capable of inhibiting the visible bacterial growth was defined as the MIC. To determine minimum bactericidal concentrations (MBCs), 10 μL were collected from each well that contained no bacterial growth and were plated on the BHI agar. The MBC was recorded as the lowest concentration capable of inhibiting bacterial growth on the agar surface after 24 h of incubation at 37 °C. All experiments were performed in triplicate, with three independent repeats.

### 4.7. Kinetic Growth Assay

The growth curve assay was performed for all bacterial strains in 96-well polystyrene microtiter plates, as described by Field et al. (2010), with modifications [80]. The bacterial suspension adjusted (10^6^ CFU/mL) and EOOG at sub-inhibitory concentration (1/2MIC) (1:1, *v*/*v*) was added to each well, and the kinetic growth was determined over a time course. Optical density (OD_620_) was measured every hour to evaluate bacterial density for a period of 24 and 18 h for *S. aureus* and *E. coli* strains, respectively. The growth level was quantified by the area under the curve (AUC), which was calculated as a metric absorbance (620 nm) distribution as a function of time. For each condition, an average value from six replicas was given. It is important to highlight that experimental protocols were carried out with selected strains from each bacterial species with a multiresistance profile (5B and P12).

### 4.8. Checkerboard Assay

The combination activity of antimicrobial compounds was determined by checkerboard assay in 96-well microtiter plates [81]. The combinations used were as follows: EOOG + OXA/CIP for *S. aureus* (5B) and EOOG + CIP for *E. coli* (P12). First, serial two-fold dilutions of drugs were prepared with BHI broth separately. Using a 96-well microtiter plate, 50 μL of each substance (1:1 *v*/*v*) were added in rows (EOOG), in ascending concentrations, and the antibiotics (CIP or OXA) were similarly distributed among the columns. Thus, each well held a unique combination of concentrations of the two substances. Afterward, 100 μL of bacterial inoculum (10^6^ CFU/mL) were added to the wells and incubated at 37 °C for 24 h. The analysis of the interaction results was performed by the fractional inhibitory concentration index (FIC*i*), defined as the sum of the MIC of the combined substances divided by the MIC of the isolated substances and and categorized as: Synergism (FIC*i* ≤ 0.5), additive (FIC*i* > 0.5 to ≤ 1), indifferent (FIC*i* > 1 to < 2), or antagonism (FIC*i* ≥ 2), according to European Committee for Antimicrobial Susceptibility Testing (EUCAST) [82].

### 4.9. Antibiofilm Activity

The EOOG antibiofilm assay was verified on preformed biofilms, 12 and 24 h for *E. coli* (P12) and *S. aureus* (5B), respectively, using 96-well polystyrene microtiter plates, according to Yadav et al. (2015), with modifications [69]. After biofilm formation, the wells were washed three times with PBS (Phosphate-Saline Buffer 0.1 M; pH 7.4) to remove the planktonic and weakly attached cells. Afterward, the biofilm was treated with 200 µL of essential oil at decreasing concentrations (8000 to 1000 µg/mL) for 4 h at 37 °C. The biofilm was quantified according to biomass and the number of viable cells by crystal violet staining (CV) and colony forming units (CFU), respectively. Each technique was performed in triplicate, with three independent experiments.

Firstly, 200 μL of methanol PA (Dinâmica, São Paulo, Brazil) were added to treated biofilm wells for 15 min to fix the adhered cells. Afterward, it was removed, and the microplate was left to dry at room temperature. Thus, 200 μL of 0.1% crystal violet (Synth^®^, Sao Paulo, Brazil) were added over a period of 10 min. After washing the wells using distilled water, the biofilm-bound dye was solubilized in 200 μL of 33% glacial acetic acid (Dinâmica, Sao Paulo, Brazil). After that, the absorbance was measured using an optical density reader (SpectraMax^®^ Paradigm^®^ Molecular Devices, San Jose, California, USA) at 590 nm. Secondly, to determine the viable number of cells in the EOOG-treated biofilm, 200 µL of PBS were added to each well, and the microplate was subjected to ultrasound (GNATUS, São Paulo, Brazil) for 5 min to detach the biofilm-embedded bacteria. Serial ten-fold dilutions were then prepared from that cell suspension, and 10 μL were plated in BHI agar. After 18 h of incubation at 37 °C, the total of enumerated cells was expressed in log_10_ CFU/mL from the average of the number of CFUs of three different wells from the same replicate.

### 4.10. Scanning Electron Microscopy

Structural arrangement changes of *S. aureus* (5B) and *E. coli* (P12) biofilms induced by EOOG (1xMIC) treatment were evaluated by scanning electron microscopy (SEM—Inspect S50—FEI Company^®^, Oregon, USA), according to Yadav et al. (2015), with modifications [69]. The biofilm was formed from a cell suspension (10^6^ cells/mL) in fresh BHI for 24 h (5B) and 12 h (P12) at 37 °C in 24-well plates containing glass slides (1 × 1 cm). The slides were then washed three times with 0.1 M PBS, and surface adsorbed cells were treated with EOOG at MIC values against 5B and P12, 2000 and 1000 µg/mL, respectively, for 4 h at 37 °C. For the control group, only fresh BHI with tween 80 was used. Before SEM analysis, the cells were pre-fixed with 2% glutaraldehyde, dehydrated with an alcohol solution at 10, 30, 50, 70, 90, and 100% for 20 min each, and dried at room temperature. The slides were placed on carbon tape on the aluminum sample holder (stubs), coated with gold (Emitech Q150T, Lewes, UK), and viewed via SEM at 20 kW.

### 4.11. Statistical Analysis

Antimicrobial and antibiofilm activity assays performed in triplicate were presented as the mean ± standard deviations (SD). Data were statistically analyzed using GraphPad Prism 8.0 software (GraphPad Software, Inc., San Diego, CA, USA) applying analysis of variance (ANOVA) with Tukey’s post hoc test. Differences between treated and control groups were considered significant when *p* < 0.01. The graphs generated in the growth curve test were used to determine the area under the curve (AUC).

## 5. Conclusions

The EO from dried leaves of *O. gratissimum* L. obtained in Maranhão State, Brazil, demonstrated antibacterial activity against representative Gram-negative and -positive strains, *S. aureus* and *E. coli*, with a multidrug resistance profile. In addition, the ability of EO combined with clinical antibiotics to increase the sensitivity of *S. aureus* to OXA and CIP was determined. It was possible to observe the action against established biofilms as well as the reduction in bacterial load within *S. aureus* and *E. coli* biofilms. Considering all the results obtained, the EOOG can be used as an alternative for new therapies against MDR bacteria, for instance, like formulations of antibacterial creams or gels containing OEs for topical applications or even to produce EO-based wound dressings.

## Figures and Tables

**Table 1:** Chemical composition from the essential oil (EO) of the *Ocimum gratissimum* L. (EOOG) leaves.

Peak	Compounds ^a^	Chemical Class	KIL ^b^	KIC ^c^	%
1	α-Pinene	HM ^d^	939	943	0.08
2	Sabinene	HM	975	982	0.17
3	β-Pinene	HM	979	986	0.43
4	Myrcene	HM	990	995	0.14
5	1,8-Cineole	OM ^e^	1031	1040	15.16
6	(*E*)-β-Ocimene	HM	1050	1053	0.10
7	Linalool	OM	1096	1103	0.34
8	δ-Terpineol	OM	1166	1174	0.12
9	Terpinen-4-ol	OM	1177	1184	0.16
10	α-Terpineol	OM	1188	1196	0.31
11	Eugenol	PH ^f^	1359	1365	74.83
12	(*E*)-Caryophyllene	HS ^g^	1419	1427	2.20
13	α-Humulene	HS	1454	1461	0.32
14	γ-Muurolene	HS	1479	1488	0.51
15	β-Selinene	HS	1490	1493	2.82
16	α-Selinene	HS	1498	1501	0.85
17	7-Epi-α-selinene	HS	1522	1525	0.26
18	Spathulenol	OS ^h^	1578	1584	0.07
19	Caryophyllene oxide	OS	1583	1590	0.55
	**Total**				**99.42**

^a^ Compounds ordered by their elution from an HP-5MS column. ^b^ Kovats indices from the literature [32]. ^c^ Kovats indices calculated against n-alkanes (C_9_–C_30_) on an HP-5MS column. ^d^ Hydrocarbon monoterpenes. ^e^ Oxygenated monoterpenes. ^f^ Phenylpropanoid. ^g^ Hydrocarbon sesquiterpenes. ^h^ Oxygenated sesquiterpenes.

**Table 2:** Antibacterial activity of the EOOG against *S. aureus* and *E. coli* strains by the paper disk diffusion test and the microdilution method.

***Staphylococcus aureus***	**IZD (mm) ^1^**	**MIC (µg/mL) ^2^**	**MBC (µg/mL) ^3^**
ATCC 6538	17	1000	1000
2B	14	1000	2000
5B	20	2000	2000
7B	15	1000	2000
***Escherichia coli***			
ATCC 11303	12	1000	1000
P12	13	1000	1000
P25	12	1000	1000
P36	13	1000	1000

Notes: IZD ^1^: Inhibition zones diameter using 6 mm disks. MIC ^2^: Minimum inhibitory concentration. MBC ^3^: Minimum bactericidal concentration.

**Table 3:** Fractional inhibitory concentrations index (FIC*i*) of OXA and CIP combined with EOOG for *S. aureus* (5B) and *E. coli* (P12) microorganisms.

Microorganisms	Combination	MIC (µg/mL)		FIC*i*	Interpretation	Drug Reduction
		Individual	Combined			
***S. aureus* (5B)**	**EOOG**	2000	1000	0.516	Additive	2x
	**OXA**	2000	31.25			64x
	**EOOG**	2000	1000	0.562	Additive	2x
	**CIP**	62.50	3.90			16x
***E. coli* (P12)**	**EOOG**	1000	1000	2.000	Antagonistic	NR
	**CIP**	62.50	62.50			NR

Notes: NR: no reduction. OXA: oxacillin. CIP: ciprofloxacin.

**Table 4:** Antibiotic resistance profile obtained by the VITEK^®^2 system.

***Staphylococcus aureus***	**Source**	**Antibiotic Resistance**
Standard	ATCC 6538	Sensitive
2B	Soft tissues	ERT, CLIN e BZP
5B	Human blood	ERT, CLIN, CIP, NOR, MOX, BZP, OXA e RIP (I)
7B	Human blood	BZP e OXA
***Escherichia coli***		
Standard	ATCC 11303	Sensitive
P12	Fish fillet	AMP, CFL, CIP, NOR, NAL e AMC (I)
P25	Fish fillet	AMP, CFL (I) e AMC (I)
P36	Fish fillet	AMP, CFL (I) e AMC (I)

Notes: AMC: Amoxicillin/clavulanic acid. AMP: Ampicillin. BZP: Benzylpenicillin. CFL: Cefelotin. CIP: Ciprofloxacin. CLIN: Clindamycin. ERT: Erythromycin. MOX: Moxifloxacin. NAL: Nalidixic acid. NOR: Norfloxacin. OXA: Oxacillin. (I): Intermediate resistance.
